# Etiological study of enteric viruses and the genetic diversity of norovirus, sapovirus, adenovirus, and astrovirus in children with diarrhea in Chongqing, China

**DOI:** 10.1186/1471-2334-13-412

**Published:** 2013-09-03

**Authors:** Zengzhi Ren, Yuanmei Kong, Jun Wang, Qianqian Wang, Ailong Huang, Hongmei Xu

**Affiliations:** 1 Department of Infectious diseases and Gastroenterology, Children’s Hospital of Chongqing Medical University, No. 136 Zhongshan Er Road, Yuzhong District, Chongqing 400014, China; 2 Key Laboratory of Developmental Diseases in Childhood, (Chongqing Medical University), Ministry of Education, No. 136 Zhongshan Er Road, Yuzhong District, Chongqing 400014, China; 3 Key Laboratory of Pediatrics in Chongqing (CSTC2009CA5002), No. 136 Zhongshan Er Road, Yuzhong District, Chongqing 400014, China; 4 Key Laboratory of Infectious Diseases, Ministry of Education, No. 1 Yixueyuan Road, Yuzhong District, Chongqing 400016, China

## Abstract

**Background:**

Enteric viruses are a major cause of diarrhea in children, especially those <5 years old. Identifying the viral agents is critical to the development of effective preventive measures. This study aimed to determine the prevalence of common enteric viruses in children <5 years old presented with diarrhea to the Children’s Hospital of Chongqing Medical University.

**Methods:**

Five hundred fecal samples were collected between August and November 2010 from children <5 years of age who presented with acute diarrhea at the Children’s Hospital of Chongqing Medical University. All samples were tested for rotaviruses A, B, and C, noroviruses GI and GII, adenovirus, sapovirus, and astrovirus using enzyme-linked immunosorbent assay, reverse transcription-polymerase chain reaction (RT-PCR), or PCR. Partial sequences of norovirus, sapovirus, adenovirus, and astrovirus were phylogenetically analyzed to determine the genotype.

**Results:**

Enteric viruses were detected in 302 of the 500 children who presented with acute diarrhea (277/477; 58.07%) and persistent diarrhea (5/23; 21.74%). In 277 samples from children with acute diarrhea in whom at least one viral agent was found, rotavirus A was the most frequent virus identified (132 cases; 27.67%), followed by norovirus GII in 130 cases (27.25%), adenovirus in 30 cases (6.29%), sapovirus in 9 cases (1.89%) and astrovirus in one case (0.21%). Twenty-two of the norovirus GII-positive cases were randomly selected for genotyping. GII/4 was the predominant strain, followed by GII/6, GII/2, GII/3, and GII/7. Sapovirus was classified into four genotypes: GI/1 was predominant, followed by GI/2, GII/1, and GIV. The predominant adenovirus was type 41. Mixed infections were found in 25 cases, all of which presented with acute diarrhea (25/477; 5.24%). Viruses were positive in 5/23 (21.74%) cases with persistent diarrhea. Neither rotavirus B, rotavirus C, nor norovirus GI were found in any of the samples.

**Conclusions:**

Enteric viruses are a major cause of diarrhea in children <5 years old in Chongqing. Rotavirus A is the most common etiological agent, follow by norovirus.

## Background

Infectious diarrhea is one of the most common diseases affecting children <5 years old, leading to significant morbidity and mortality worldwide, especially in developing countries. Diarrhea causes >1.8 million deaths each year [1]. Although many pathogens can cause diarrhea, >75% of cases are caused by viruses [2]. Rotaviruses are the leading cause of severe diarrhea worldwide among children <5 years of age [3]. More specifically, rotaviruses A-C commonly infect humans, and rotavirus A predominates in children [4]. Rotavirus B and C infections have been reported to cause only sporadic cases and outbreaks [5,6]. Recent studies have reported that norovirus is the second most frequent diarrhea-causing virus [7]. Sapoviruses, astroviruses, and adenoviruses have also been reported to cause diarrhea in children [8]. However, little is known about the current prevalence of viral diarrhea-causing pathogens in children in Chongqing, a metropolitan city in western China with a population of 30 million. This study aimed to evaluate the roles of different viruses in causing acute and persistent diarrhea in children <5 years of age who presented to a university children’s hospital in Chongqing. The molecular characteristics of the noroviruses, sapoviruses, astroviruses, and adenoviruses were also determined.

## Methods

### Sample collection and viral RNA/DNA extracted

Stool samples were collected from children <5 years of age seeking medical care for acute and persistent diarrhea between August and November of 2010 in the outpatient department of the Children’s Hospital of Chongqing Medical University. Diarrhea was defined according to the WHO criteria for children [9] as the occurrence of three or more loose, liquid, or watery stools within a 24-hour period. Acute diarrhea was defined as <2 weeks’ duration and persistent if >2 weeks’ duration. All samples were stored at −70 °C until further study. Viral RNA/DNA was extracted manually using a QIAamp® Viral RNA Mini Kit (QIAGEN, Hilden, Germany) according to the manufacturer’s instructions. The study protocols were approved by the Institutional Review Board of the Children’s Hospital of Chongqing Medical University. Signed informed consent was obtained from the parent or guardian.

### Viruses detection

Rotavirus A in stool samples was detected using a Colloidal Gold device (Huian, Shenzhen, China) according to the manufacturer’s instructions. Rotaviruses B and C, noroviruses GI and GII, sapovirus, and astrovirus were detected using reverse transcription-polymerase chain reaction (RT-PCR). Adenovirus was tested using PCR. For RT, the viral RNA was reverse transcribed using the SuperScript® III First-Strand Synthesis System for RT-PCR (Invitrogen, Carlsbad, CA, US) according to the manufacturer’s instruction. PCR was conducted as described previously [10-12].

### Genotyping of norovirus, sapovirus, adenovirus, and astrovirus

The PCR products were purified using a PCR Purification Kit following the manufacturer’s instructions (Sangon Biotech Co. Ltd, Shanghai, China). PCR primers were also used for the DNA sequencing. The DNA sequencing was performed in an Applied Biosystems DNA Sequencer (model 3730XL) at the Sequencing Center of Chongqing Medical University. The obtained sequences were compared to the reference strains using BLAST searches. Sequence data from this article have been deposited with the Gene Bank Libraries under accession numbers KF495121–KF495182 and KF512009. Phylogenetic trees were constructed using the MEGA program (version 5.05) by the neighbor-joining method.

## Results

A total of 500 fecal samples were collected from 500 children <5 years of age. The age distributions of the patients of this study were grouped in five-year segments: 0–5, 6–11, 12–23, 24–35, and 35–60 months of age. In this study, 93.60% of all infants were <2 years old. Of the 500 cases, 477 cases were acute diarrhea and 23 cases were persistent diarrhea. For cases with persistent diarrhea, the duration was 14–21 days in 20 cases, 22–28 days in two cases, and exceeded 60 days in only one case.

Rotavirus A was identified in 132 samples (27.67%) of 477 cases with acute diarrhea. More than 94% of the identified cases occurred in children <2 years of age. However, the frequency was low among infants aged 0–5 months, increased and peaked in children aged 6–23 months, and declined among children >24 months (Table 1). Of the 23 cases of persistent diarrhea, rotavirus A was detected in two cases aged 7 months and 10 months old, a positive rate of 8.70% (2/23). Neither rotavirus B nor C was found in this study.

**Table 1:** Distribution of viral agents in 477 children with acute diarrhea in different age groups in Chongqing

**Age group**	**No. of**	**Rotavirus A**	**Norovirus GII**	**Sapovirus**	**Adenovirus**	**Astrovirus**	**Mixed infection**
**(months)**	**sample**	**n (%)**	**n (%)**	**n (%)**	**n (%)**	**n (%)**	**n (%)**
<6	102	18 (13.63)	16 (12.31)	1 (11.11)	4 (13.33)	1 (100%)	4 (16.00)
6–11	221	55 (41.67)	76 (58.46)	2 (22.22)	12 (40.00)	0	10 (40.00)
12–23	122	54 (40.91)	29 (22.31)	6 (66.67)	9 (30.00)	0	7 (28.00)
24–35	16	2 (1.52)	7 (5.38)	0 (0)	1 (3.33)	0	2 (8.00)
36–60	16	3 (2.27)	2 (1.54)	0 (0)	4 (13.33)	0	2 (8.00)
Total	477	132	130	9	30	1	25

**Figure 1:**
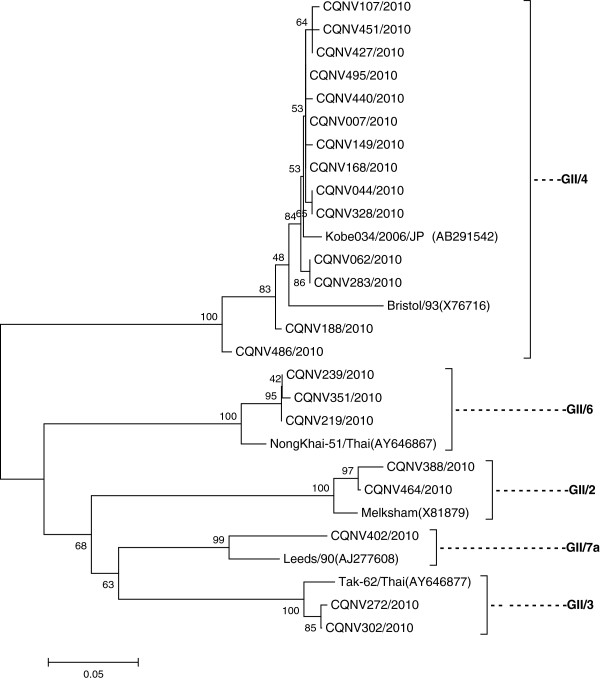
**Phylogenetic trees constructed from partial nucleotide sequences of the capsid gene of norovirus GII.** The calibration scale indicates the percent divergence among the nucleotide sequences. The reference sequences were obtained from GenBank.

Norovirus GII was detected in 130/477 (27.25%) samples of acute diarrhea. The highest frequency (58.46%) was found in infants aged 6–11 months, followed by children aged 12–23 months at a frequency of 22.31% (Table 1). Norovirus GII was found in two children aged 4 and 5 months with persistent diarrhea, a positive rate of 2/23 (8.70%). Twenty-two PCR products that were selected randomly from 132 norovirus GII-positive samples were sequenced and classified into five distinct genotypes, including GII/4 (14/22; 63.64%), GII/6 (3/22; 13.63%), GII/2 (2/22; 9.09%), GII/3 (2/22; 9.09%), GII/7 (1/22; 4.55%) (Figure 1). No cases of norovirus GI were identified in this study.

**Figure 2:**
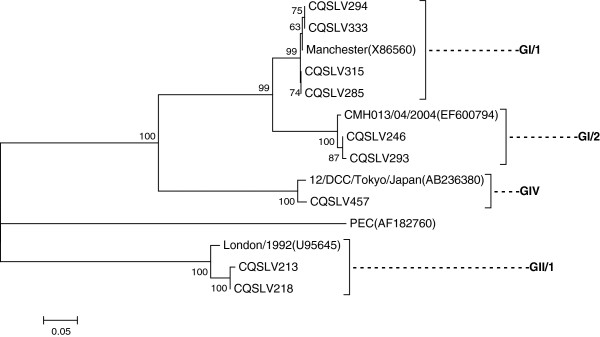
**Phylogenetic trees constructed from partial nucleotide sequences of the capsid gene of sapovirus.** The calibration scale indicates the percent divergence among the nucleotide sequences. The reference sequences were obtained from GenBank.

Nine stool samples were positive for sapovirus, all of which (9/477, 1.89%) were from children <24 months of age with acute diarrhea (Table 1). Nine sapovirus strains were classified into three distinct genogroups, GI (6/9; 66.67%), GII (2/9; 22.22%), and GIV (1/9; 11.11%). Six strains of GI were further classified into two genotypes: four strains were GI/1 and two strains were GI/2. The two GII strains were GII/1 (Figure 2).

**Figure 3:**
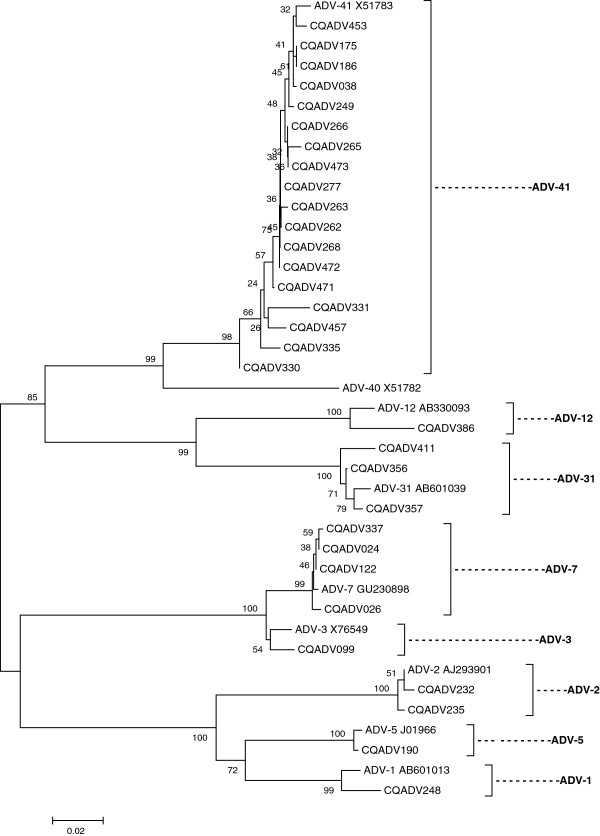
**Phylogenetic trees constructed from partial nucleotide sequences of the hexon gene of adenoviruses.** The calibration scale indicates the percent divergence among the nucleotide sequences. The reference sequences were obtained from GenBank.

In this study, 31 stool samples tested positive for adenovirus. Thirty positive samples (30/477; 6.29%) were associated with acute diarrhea with a high frequency in infants aged 6–23 months. And 25/30 (83.33%) of adenovirus-positive stool samples were from children <2 years of age (Table 1). Adenovirus was detected in a child aged 12 months who had diarrhea for 25 days. Genotyping analysis showed the presence of type 41 (18/31; 58.06%), type 7 (4/31; 12.90%), type 31 (3/31; 9.68%), type 2 (2/31; 6.45%), type 1 (1/31; 3.23%), type 3 (1/31; 3.23%), type 5 (1/31; 3.23%), and type 12 (1/31; 3.23%) adenoviruses (Figure 3).

Only one sample (1/477; 0.21%) in a child with acute diarrhea was positive for astrovirus. The amplified PCR product was sequenced and 98% nucleotide sequence identity was found with human astrovirus strain HCMC220/2006 (EU030292) belonging to astrovirus type 1.

Mixed infections were found in 25 cases, all of which were acute diarrhea (25/477; 5.24%). Eleven of the mixed infection cases were due to dual infections. The most common dual infections were rotavirus A combined with other viruses, accounting for 18/25 (72.00%) cases including 11 combined with norovirus GI and seven combined with adenovirus. Norovirus GII combined with adenovirus was detected in five cases, while norovirus GII combined with astrovirus and adenovirus combined with sapovirus were detected in one case each.

The results of this study showed that at least one viral agent was found in 277/477 (58.07%) cases of acute diarrhea and 5/23 (21.74%) cases of persistent diarrhea. Single-virus cases of acute diarrhea included rotavirus A in 114/477 (23.90%), norovirus GII in 113/477 (23.69%), adenovirus in 17/477 (3.56%), and sapovirus in 8/477 (1.67%). Two or more viral infections in each case were found in 25/477 (5.24%). Viruses were detected in 5/23 cases of persistent diarrhea, including rotavirus A in two cases, norovirus GII in two cases, and adenovirus in one case.

## Discussion

Diarrheal disease is a major cause of worldwide mortality in children. Viruses have been recognized as a primary cause of childhood diarrhea. In our study of 500 children’s stool samples, we found positive virus rates of 277/477 (58.07%) in acute diarrhea and 5/23 (21.74%) in persistent diarrhea. Our findings confirmed our clinical impression that viruses are the major cause of acute diarrhea in Chongqing.

Rotavirus A was detected in 27.67% cases of acute diarrhea. This finding indicates that rotavirus A is the most common etiologic agent of acute diarrhea in children even though the period of study—August to November—is the high epidemic season for norovirus [13]. Consistent with the earlier study, the highest frequency of rotavirus A infection was in children 6–23 months old and >96.0% occurred among children <2 years of age [14]. Therefore, a rotavirus vaccine should be widely introduced to reduce the morbidity of diarrhea in children in this area. No cases of rotavirus B or C were detected in this study, indicating that they are not the pathogens causing diarrhea in children <5 years old in Chongqing. Other studies have reported they were frequently detected in stools of older children and adults with diarrhea [15,16].

The positive rate of norovirus detection was very close to that of rotavirus, especially in acute diarrhea. Therefore, norovirus also plays a very important role in acute diarrhea in younger children in Chongqing. All detected noroviruses were GII, a finding that was concordant with those of two other epidemiological studies conducted in Hong Kong [17] and Japan [10]. GII/4 was the most predominant genotype (63.64%) in this study, a finding that was concordant with those of other studies in countries such as Thailand, Japan, and USA [18-20].

Nine stool samples, all of which were from cases of acute diarrhea (9/477; 1.89%), were positive for sapovirus. This result was consistent with those of published reports that showed that its prevalence is usually much lower than norovirus [17,21]. GI was found to be the most common genogroup, with GI/1 (66.67%) the most predominant strain, followed by the GI/2, GII/1, and GIV strains. Some studies showed that the detection rate of sapovirus strains circulating in several regions worldwide varied over time; nevertheless, GI was the predominant sapovirus strain [22-24].

The positive adenovirus rate of in the current study was 6.29% in acute diarrhea, a finding that is similar to that of a report from other parts of China [14]. Our results suggest that adenovirus is also an etiologic agent in young children with acute diarrhea in Chongqing. Enteric type 41 adenovirus was the most prevalent (58.06%). Other genotypes, such as type 7, type 31, type 2, type 1, type 3, type 5, and type 12, which are generally known causes of acute respiratory disease in children, were also detected in this study. Our result is similar to those of other reports in India, Brazil, and South Korea [25-27]. The mechanisms of these adenoviruses in diarrhea should be further investigated.

Only one of the stool samples in this study was positive for astrovirus, suggesting that this virus is rare in Chongqing. However, one study detected a prevalence rate of 5.5% for astrovirus in seven provinces of China [28]. The samples in our study were not collected during the peak astrovirus infection season (October–January) [28]. Hence, it may be possible that the current study missed the peak season of this virus. Therefore, further work is required to determine the prevalence of astrovirus in this pediatric Chongqing population during its peak season.

The systematic detection of the five viruses in the current study allowed us to observe a relatively high proportion of co-infection at a rate of 5.24% in children with acute diarrhea. All of the mixed infections were dual in nature, especially between rotavirus A and norovirus GII. This finding is consistent with the results of earlier studies [2,29].

Of the 23 samples of children with persistent diarrhea, five were positive for the viruses detected in this study, including two for rotavirus A, two for norovirus GII, and one for adenovirus. The duration of diarrhea was <1 month. The causes of persistent diarrhea are complicated. A virus and/or secondary lactose intolerance caused by a viral infection could be a cause of persistent diarrhea.

There was a limitation in this study. The 500 samples in this study were collected from August to November, 2010. This period is short to describe characteristics of epidemiology of viral diarrhea in Chongqing. It was just a preliminary study to determine common enteric viruses in children ≤5 years old with diarrhea in this area. On the base of this study, the epidemiology of enteric viruses will be studied further.

## Conclusions

The results of this study show that viruses are the main cause of acute diarrhea in Chongqing, China. Rotavirus and norovirus are the two predominant viruses. Sapovirus, adenovirus, and astrovirus are responsible for only a small percentage of children with acute diarrhea. However, our study is limited by the season during which the samples were collected.

## Competing interests

The authors declare that they have no competing interests.

## Authors’ contributions

Authors contributed to the presented work as follows. XH and HA designed the study. RZ carried out the sample collection, sample testing, statistical analysis and drafted the manuscript. KY, WJ and WQ contributed to sample collection. KY also contributed to the drafting of the manuscript. All authors read and approved the final manuscript.

## Pre-publication history

The pre-publication history for this paper can be accessed here:

http://www.biomedcentral.com/1471-2334/13/412/prepub
